# Development and theoretical investigation of antimony-based halide perovskite solar cell using kesterite as hole transport material

**DOI:** 10.1038/s41598-025-27168-6

**Published:** 2025-12-09

**Authors:** Chinedu Christian Ahia, Nicholas Rono, Edson Leroy Meyer

**Affiliations:** Fort Hare Institute of Technology, University of Fort Hare, Private Bag X1314, Alice, 5700 South Africa

**Keywords:** Perovskite, Charge carrier, Recombination, Efficiency, Photovoltaics, Cesium antimony iodide, Energy science and technology, Materials science, Physics

## Abstract

Cesium antimony iodide (Cs₃Sb₂I₉) is a lead-free halide perovskite attracting significant attention due to its tunable bandgap, unique optoelectronic properties, and potential as a light-absorbing material in perovskite solar cells (PSCs). Kesterite CZTSe, a stable, non-toxic, and cost-effective material that facilitates efficient hole extraction, reduces recombination, and is compatible with lead-free perovskites, is employed in this study as a novel hole transport layer (HTL) for high-performance, sustainable solar cells. The influence of various parameters on the device’s performance with the general architecture of FTO/WS_2_/Cs_3_Sb_2_I_9_/CZTSe/Ag was investigated theoretically using SCAPS-1D numerical software. Optimization of the device yielded a notable power conversion efficiency (PCE) of 21.02% and a high fill factor (FF) of 81.94%, underpinned by a type-II band alignment that promotes efficient charge transport. The simulated quantum efficiency exceeded 95% across 300–650 nm, demonstrating excellent visible-light absorption. Temperature-dependent analysis indicated stable operation within 240–320 K, while open-circuit voltage (V_oc_) increased sharply with acceptor density above 10²⁰ cm⁻³, reflecting enhanced built-in potential and reduced recombination. The results contained in this study are projected to advance knowledge towards the understanding of complex phenomena in emerging materials and technologies that could be beneficial for performance optimization in solar devices, thus, their future commercialization.

## Introduction

Economic and environmental pressures, including climate change and greenhouse gas emissions, are driving the shift to renewable energy. Wind and solar offer sustainable, low-emission alternatives to finite fossil fuels, reducing pollution and protecting ecosystems and public health. Solar energy has been recognised as an appropriate substitute for fossil fuel^1^ due to its abundant supply and clean form. Hence, ongoing efforts are directed toward developing sustainable approaches for optimizing the power conversion efficiencies (PCE) of solar cells. The high price of silicon-based solar panels, which currently control the majority of the market^2^, the comparatively low conversion power output, and the intricate production processes that require advanced machinery and technology^3^ are some of the remaining obstacles in solar harvesting and conversion strategies. Therefore, effective methods that enable the sustainable harvesting and use of sunlight must be developed.

Antimony-based perovskites have gained attention for their excellent optoelectronic properties, low toxicity, enhanced stability, and established fabrication techniques, making them promising light-absorbing layers in solar cells with performance comparable to lead-halide perovskites^4^. Furthermore, it has been discovered that adding inorganic Cs_+_ cations to the active layer in place of organic cations can increase the stability of Pb-free antimony-based perovskites. As a result, antimony is thought to be a substitute for lead in metal halide perovskites^5^. Likewise, first principles calculations to investigate the optoelectronic properties of cesium antimony iodide (Cs_3_Sb_2_I_9_) revealed its suitability as a perovskite material for solar cells^5^. Studies on synthesized colloidal Cs_3_Sb_2_I_9_ nanocrystals^6^ indicated that they revealed 2D crystal structures and a remarkable absorption cross-section of about 10^15^cm^2^, distinguishing them as prospective materials for optoelectronic applications^7^.

A two-step deposition technique was used by Saparov et al. in 2015 to create a thin film of big particle layered Cs_3_Sb_2_I_9_, which was observed to exhibit more stability compared to MAPbI_3_ perovskite when subjected to ambient conditions^8^. The lead-free antimony perovskite-inspired absorber Cs₃Sb₂I₉ exhibits a wide bandgap of ~ 2.05 eV^8^, strong absorption coefficients exceeding 10⁵ cm⁻¹^8^, and favorable ambient stability^9^. However, its device power conversion efficiencies (PCEs) remain low, with reported champion values of only ~ 2.5%^9^. In contrst, the all-inorganic lead halide CsPbI₃ offers a more suitable bandgap (~ 1.68–1.74 eV for α/γ phases^10,11^ and has achieved state-of-the-art efficiencies up to ~ 21.1% under stabilized condtions^12^, though its black phase requires stabilization strategies to ensure long-term stability^12^. The tin analogue CsSnI₃ has the most favorable bandgap (~ 1.2 eV^13^ for single-junction photovoltaics and has recently demonstrated PCEs up to ~ 12%^14^, but suffers from intrinsic instbility due to Sn(II) oxidation^13,14^. Lead-free double perovskites such as Cs₂AgBiBr₆ exhibit indirect, wide bandgaps of ~ 1.9–2.2 eV^15^, limiting current densities and resulting in modest PCEs (≤ 6%^15^, though with excellent stability and non-toxiciy. Similarly, the bismuth analogue Cs₃Bi₂I₉ has a wide indirect bandgap of ~ 2.0–2.3 eV^15^, with reported solar cell efficiencies restricted to the low single-digit range^15^. Overall, while lead-free alternatives such as Cs₃Sb₂I₉, CsSnI₃, and double perovskites offer improved stability and reduced toxicity, they still lag significantly behind Pb-based CsPbI₃ in terms of efficiency. Boopathi et al. discovered in 2017 that Cs_3_Sb_2_I_9_ had a band gap of 2.0 eV and good light absorption characteristics^16^, while in 2019, Umar et al. considerably controlled the crystallization by the anti-solvent procedure, which decreased the trap state density of antimony-based perovskite and allowed the device FTO/TiO_2_/Cs_3_Sb_2_I_9_/Au to reach a PCE of 1.21%^17^. By adding thiourea to the precursor solution, Li et al. controlled the crystallization orientation of Cs_3_Sb_2_I_9 − x_Cl_x_ in 2022, which successfully encouraged carrier transport and caused perovskite solar cells to exhibit good stability^18^. Based on the reviewed studies, Cs₃Sb₂I₉ shows strong potential as a candidate material for future perovskite solar cells.

Furthermore, kesterites are a class of entirely inorganic materials that are appealing for the large-scale development of thin-film photovoltaic (PV) technology because of their earth-abundance, natural p-type conductivity, excellent absorption coefficient, direct band gap energy that can be modified between 1.0 and 1.6 eV by altering the elemental composition, and low material consumption^19^. Until now, the best efficiency for pure sulfide CZTS, which has a higher bandgap, is 11.4%, while the record efficiency for selenium-treated kesterite, which has a low bandgap, is 14.9%^20^. To improve the performance of kesterite-based solar cells, which is required for industrial implementation, several research groups have made major efforts^21–26^. A recent study^27^ has demonstrated that Cd-free kesterite thin-film solar cells have the potential to become the material of choice for environmentally friendly energy production in the context of newly developed photovoltaics.

In essence, kesterite Cu_2_ZnSnSe_4_ (CZTSe), known as copper zinc tin selenide, is an emerging chalcogenide material that has garnered more attention in recent years and is considered favourable for photovoltaic technology applications owing to its thermodynamic stability^28^, earth-rich and eco-friendly components, suitable bandgap (1.0–1.5 eV), and ample light absorption coefficients (> 10^4^ cm^− 1^). According to an investigation on cost analysis^29^, the abundance of raw materials utilized in CZTS thin-film technology gives it a significant manufacturing cost advantage over copper indium gallium selenide (CIGS) thin-film technology. Moreover, CTZS and CTZSe have been considered as suitable alternatives to substitute conventional organic hole transport layers such as spiro-OMeTAD and PEDOT: PSS in perovskite solar cells^30,31^. This is as a result of its extraordinary electrical properties, robust stability, simple fabrication technique, adjustable band, and elemental ampleness.

No existing solar material simultaneously achieves high absorption, stability, low cost, non-toxicity, and abundance. Flexible ultra-thin crystalline Si solar cells^32^, thin-film Si tandem solar cells^33^, flexible chalcopyrite CuInGa(S, Se)2 (CIGSSe) thin-film solar cells, and organic solar cells^34,35^ are the primary commercialized photovoltaic technologies at the moment. Despite their extremely high PCE, flexible III-V solar cells are only utilized in space and military applications because of their prohibitively high manufacturing costs^36^. Limitations of crystalline Si (brittleness, high cost) and organic solar cells (stability) drive the search for sustainable materials with optimized PCE for next-generation photovoltaics. The performance of a cesium antimony iodide solar cell was simulated and optimized using SCAPS-1D, examining factors such as defect density, operating temperature, and dopants in the ETL/HTL with respect to key photovoltaic metrics. This study explores non-toxic, efficient, and cost-effective materials for Pb-free perovskite solar cells, aiming to reduce recombination, enhance hole transport, and guide the design of sustainable device configurations. The theoretical results provide a foundation for experimental validation and prototyping of the proposed device.

## Methodology and proposed device configuration

In recent times, the role of numerical simulation in PSC device research has grown significantly. This can be attributed to the effectiveness of theoretical simulation of solar cells, which offers advanced knowledge on the principal operational mechanism of these devices and permits flexible modifications of various cell components, such as the density of defects in the absorber, among other factors. As a result, this approach saves time and resources, cuts down on the expenses associated with experiments, serves as a reliable platform for virtual prototyping, scalability analysis, quality control, and provides access to detailed information that might be inaccessible if relying solely on experimental methods. Numerical simulations were conducted using the Solar Cell Capacitance Simulator in 1-Dimension (SCAPS-1D) software, which was created by Professor Bulgeman and his team at Belgium’s University of Ghent. SCAPS-1D software is extensively used due to its intuitive interface, which is adaptable and offers accurate modelling functionalities for a range of thin-film solar cell designs, making it an essential tool for analysing and improving device performance. Given specified input parameters, the software can solve definite coupled equations (such as the continuity and Poisson’s equations) using numerical methods to extract information, some of which includes the electron and hole concentrations, current densities, electrostatic potential, and electric field distribution within the device. Output files containing the simulated data are generated and critically analysed to acquire a thorough understanding of the device characteristics, optimize its design, and forecast its performance attributes under different operational environments.

**Fig. 1:**
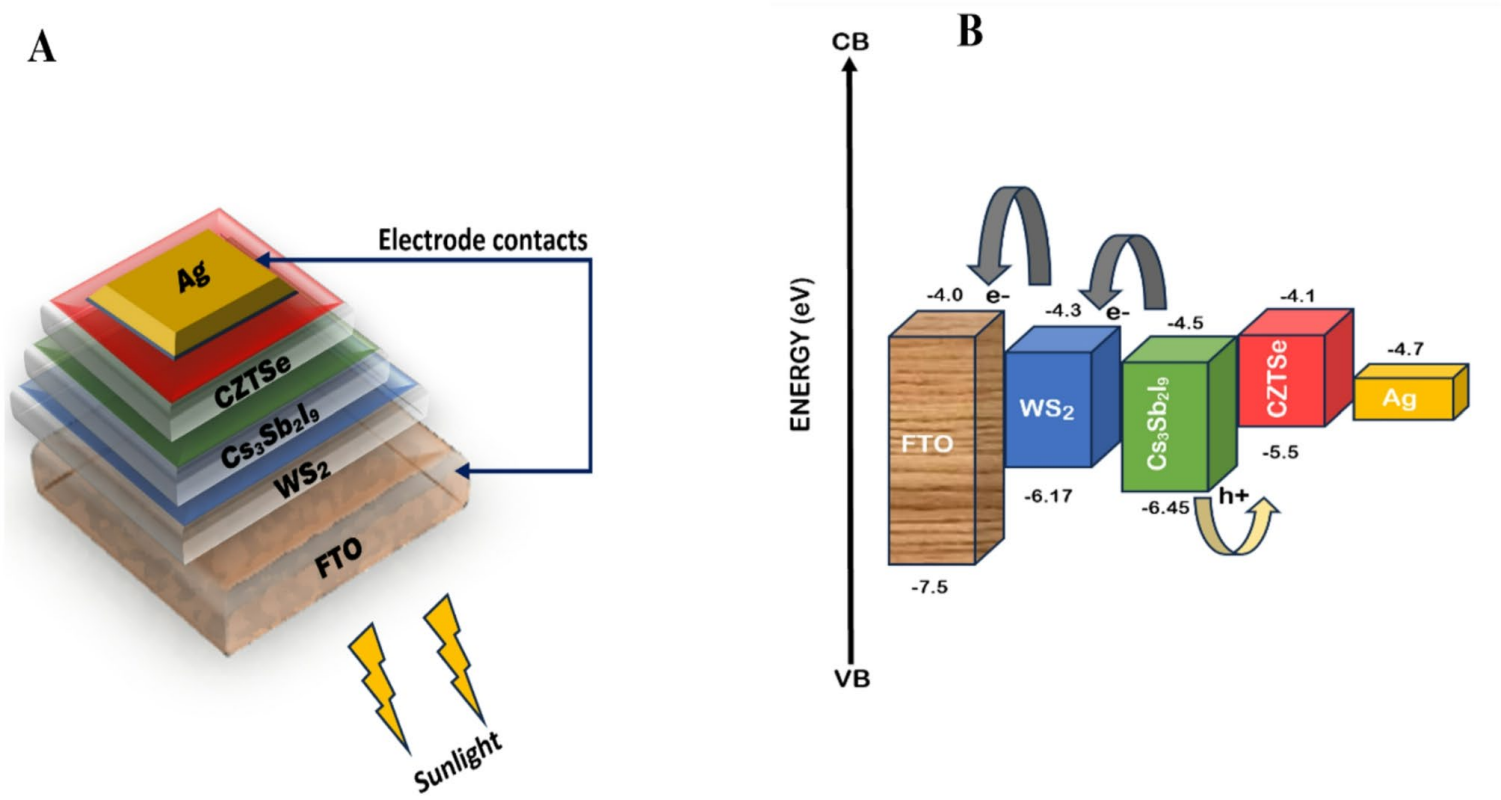
(**a**) Schematic cross-sectional representation of device structure and (**b**) the band alignment diagram.

Numerical simulation of the device with general architecture FTO/WS_2_/Cs_3_Sb_2_I_9_/CZTSe/Ag as shown in Fig. 1a, was executed using SCAPS-1D software with CZTSe as the hole transport layer (HTL), while Cs_3_Sb_2_I_9_ was used as the absorber material. The influence of various parameters on the device, such as the effect of changing the doping density of the HTL, the density of defects in the absorber layer, and the device’s operating temperature, was investigated in the present study for optimal performance of the device.

To describe the charge transfer mechanism, the energy levels of the materials utilized in the proposed device are displayed in the band alignment diagram in Fig. 1b. The principal input parameters used for the simulation of the proposed cell configuration are specified in Table 1. Also, illumination corresponding to AM 1.5 Global (AM 1.5 G), which is equivalent to 100 mWcm^− 2^ radiant power per area incident on the surface, was adopted in this study to emulate actual sunlight conditions. Other simulation conditions used include interface defect parameters displayed in Table 2. The parameters for the defect interfaces are vital in optimizing the interface materials and accounting for recombination centres at the boundary between the layers. Fluorine-doped tin oxide (FTO) serves as the front electrode in addition to being the transparent conducting oxide (TCO). Most of the light may reach the active layer with little optical loss because of its high transparency in the visible spectrum, which is normally between 85% and 90%^37^. Within the ultraviolet domain, its absorption coefficient, which extends from 10^4^ to 10^5^ cm^− 1^, is intensified, while being insignificant in the visible range^38^. Its strong electrical conductivity facilitates effective charge collection, which is essential for reaching high PCE, while its transparency guarantees less light loss.

**Table 1:** Base input parameters.

Parameters	FTO^39^	WS_2_^40^	Cs_3_Sb_2_I_9_^41^	CZTSe ^42^
Band gap, Eg, (eV)	3.50	1.87	1.95	1.40
Electron affinity, χ (eV)	4.00	4.30	4.50	4.10
Dielectric Permittivity (e_r_)	9.00	11.90	4.19	9.00
Density of states at CB, N_c_ (cm^– 3^)	2.2 × 10^18^	1.0 × 10^18^	1.0 × 10^18^	2.2 × 10^18^
Density of states at VB, N_v_ (cm^– 3^)	1.8 × 10^19^	2.4 × 10^19^	2.4 × 10^19^	1.8 × 10^19^
Electron mobility, µ_e_ (cm^2^ V^− 1^s^− 1^)	20.00	260	11.30	100
Hole mobility, µ_h_ (cm^2^ V^− 1^s^− 1^)	10.00	51	5.58	12.5
Density n-type doping, *N*_D_ (cm^– 3^)	1.0 × 10^19^	1.1 × 10^19^	0.00	0.00
Density p-type doping, *N*_A_ (cm^– 3^)	0.00	0	1.0 × 10^15^	1.0 × 10^19^
Defect density, N_t_ (cm^− 3^)	0.00	1.2 × 10^11^	1 × 10^14^	1.0 × 10^14^

Theoretically, the feasibility of replacing TiO_2_ with tungsten disulphide (WS_2_), a well-known transition metal dichalcogenide (TDMC)^43^, as an alternative electron transport layer (ETL) material in perovskite solar cells has been explored with the aim of minimizing hysteresis, boosting device performance and stability^44^. Transition metal dichalcogenides (TDMCs) are intriguing materials for optoelectronic device applications because of their distinct electrical, optical, and electrochemical characteristics^45,46^. In this work, WS_2_ is considered a promising material for electron layer application due to its inherent n-type semiconductor characteristics, suitable conductivity, extraordinary electron conduction properties, and remarkable carrier mobility and stability in dye-sensitized solar cells^47^. Additionally, the WS_2_ with the smaller band gap of 1.87 eV compared to that of the absorber of about 1.95 eV will absorb relatively more radiant energy than the absorber and thus, more photon reaching it. In the same vein, the relative semi-transparent nature of the WS_2_ material will allow effective passage of light into the device and thus better absorption which will lead to enhance PCE^48^. Likewise, silver (Ag) is a preferred choice as a back metal contact because of its combination of amiable characteristics, such as low work function, high electrical conductivity and reflectivity, strong adhesion to other materials, and its cost-effectiveness compared to other metallic alternatives like palladium (Pd), gold (Au), iridium (Ir), and platinum (Pt).

**Table 2:** Input parameters for the defect interfaces.

Interface parameter	HTL/active layer	ETL/active layer
Defect type	Neutral	Neutral
Capture cross-section electrons (cm^2^)	1.0 × 10^− 19^	1.0 × 10^− 19^
Capture cross-section holes (cm^2^)	1.0 × 10^− 19^	1.0 × 10^− 19^
Energetic distribution	Single	Single
Reference for defect energy level E_t_	Above the highest E_V_	Above the highest E_V_
Energy with respect to a reference (eV)	0.600	0.600
Interface defect (cm^− 2^)	Variable	Variable

## Results and discussions

**Fig. 2:**
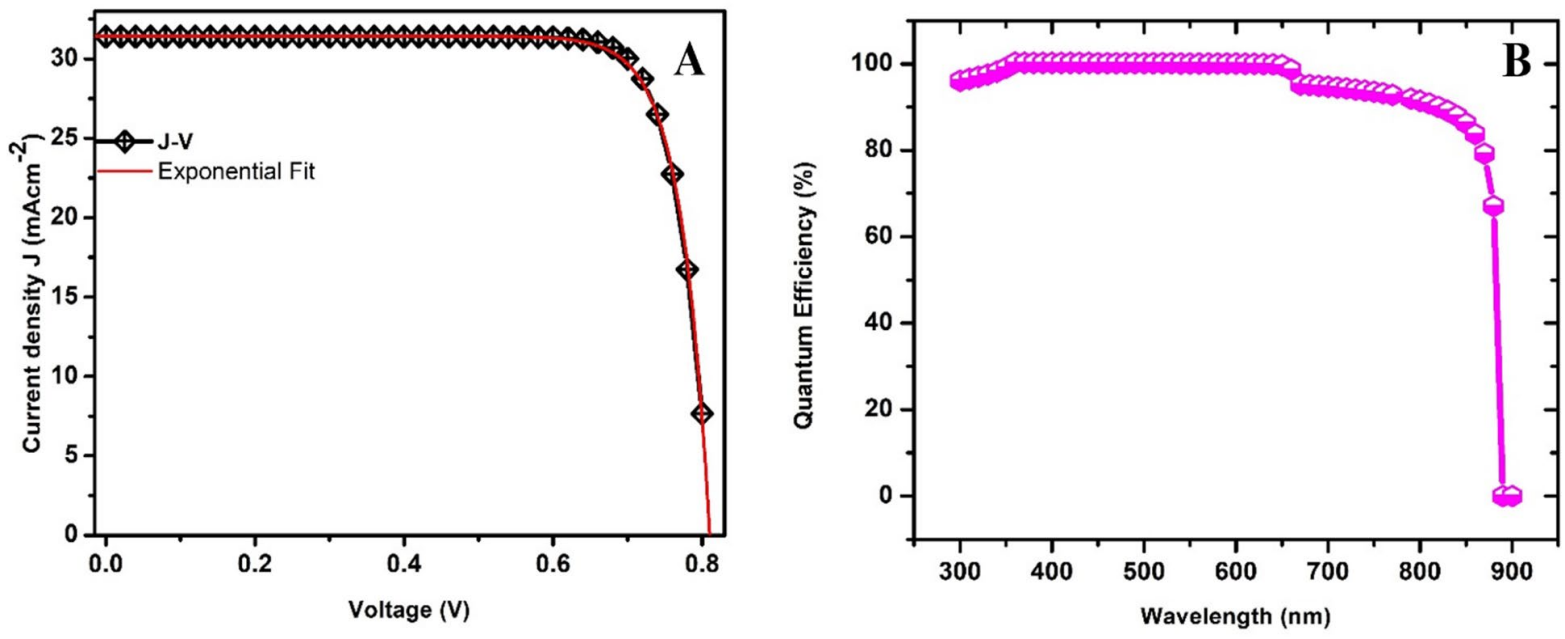
Photovoltaic performance of device (**a**) Current versus voltage and (**b**) Quantum efficiency curves.

As the initial step, thickness optimizations for all the device’s layers was carried out. While maintaining the thickness FTO, ETL absorber constant at 0.5, 2.0, and 0.20 μm, respectively, the thickness of HTL was varied from 2.8 μm to 3.8 μm and the best HTL thickness was achieved at 3.6 μm. Using the obtained HTL and still maintaining the absorber and FTO thickness, the ETL was changed from 1.5 to 2.0 μm and its’ optimized thickness was set to be 1.8 μm. Similarly, the, FTO, ETL, and HTL maintained, the absorber thickness was also changed from 0.1 μm to 0.19 μm and the ideal thickness was achieved at0.15 μm. Finally, the FTO thickness varied from 0.01 to 0.08 μm but not much changed in performance was observed. Nonetheless, it was maintained at 0.05 μm because experimentally, it is always kept constant as purchased form the manufacturer. The current density-voltage (J-V) characteristics are shown in Fig. 2a. The maximum voltage (*V*_*oc*_) the device can produce under open-circuit conditions is approximately 0.812 V and is indicated by the intersection point on the x-axis. Similarly, the maximum current (*J*_*sc*_) produced by the device is 31.40 mAcm^− 2^ and is depicted by the intersection on the y-axis. Figure 2b describes the relationship between quantum efficiency and wavelength. The results indicate that the ratio of the number of charge carriers generated to the number of photons is approximately above 95% within the wavelength range of 300 to 650 nm for the proposed device. However, the quantum efficiency begins to decrease at wavelengths above 700 nm and drops significantly after 800 nm. The slight dip in quantum efficiency around 650 nm to 700 nm, dropping from approximately 100% to 95%, could be attributed to the material’s absorption and conversion properties or other factors such as optical losses and wavelength dependency effects. The external quantum efficiency (EQE) at wavelength λ measures the fraction of incident photons that generate collected carriers. EQE dips in solar cells primarily originate from optical losses that limit the number of photons contributing to photocurrent generation^49^. Optical losses reduce the photon flux available for charge generation and therefore produce wavelength-dependent EQE dips. A convenient model that captures the main effects is:

1 $$\:EQE\left(\lambda\:\right)=\left[1-R\left(\lambda\:\right)\right]\:A\left(\lambda\:\right)\:IQE\left(\lambda\:\right)$$

where.

- R(λ)is the reflectance at the front surface (fraction of photons reflected),
- A(λ)is the fraction of incident photons absorbed in the active absorber layer, and.
- IQE(λ)is the internal quantum efficiency (probability that an absorbed photon yields a collected electron–hole pair).

Reflection at interfaces, often traced to intrinsic material properties as well as extrinsic surface or device design factors, reduces photon flux entering the absorber. Parasitic absorption in transport layers or electrodes leads to photon energy dissipation as heat rather than charge generation. In thin-film structures, interference effects between multilayers can produce destructive interference at specific wavelengths, lowering photon absorption. Furthermore, when the absorber layer is too thin, photons with longer absorption lengths pass through without being absorbed, thereby reducing electron–hole pair generation. Collectively, these optical phenomena reduce the overall carrier generation rate, manifesting as wavelength-dependent EQE dips as seen in Fig. 2b. A summary of the photovoltaic parameters obtained from the numerical simulation of the device is shown in Table 3.

The obtained PCE of 21.01% (Table 3) can be relatively higher than that of the experimental values of Sb-based perovskites which is less than a PCE of 5%. The reason for disparities between theoretical and experimental values can be explained as follows; in the simulation work, the SCAPS-1D only considers one dimensional scenario yet the actual device is in 3-dimensional (3D), thus the performance values can be higher than experimental. Also, the impact of the environmental factor such as moisture, temperature variations and the real collapse of the device structure due to excess heat or moisture is not considered. These aspects are very significant in the actual fabrication of the devices experimentally^50^. Nonetheless, the reported PCE could provide guidance on the potential enhanced performance of the device with this configuration in the future.

**Table 3:** Photovoltaic performance of device.

Parameter	
V_oc_ (V)	0.812
J_sc_ (mA cm^− 2^)	31.40
FF (%)	81.94
PCE (%)	21.02

### Influence of the density of defect of the absorber

**Fig. 3:**
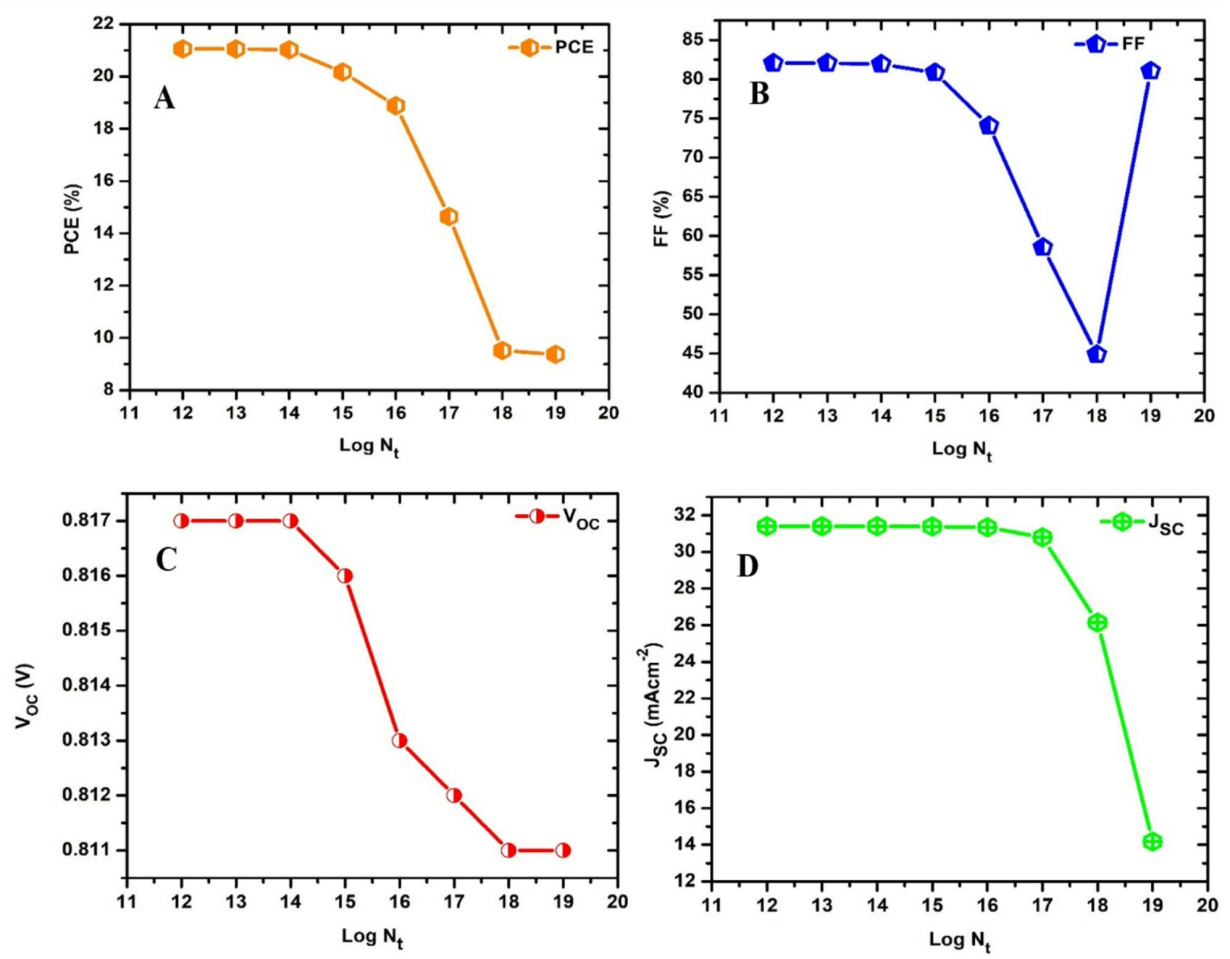
Photovoltaic performance of the best device as a function of changing the absorber density of defect (**a**) PCE, (**b**) FF, (**c**) V_oc_ and (**d**) J_sc_.

The density of defects of the absorber plays a critical role in determining the photovoltaic behaviour and performance of a solar device^44^. A defective absorber can introduce anomalies that can generate Shockley-Read-Hall (SRH) non-radiative recombination centres. These centres can disrupt the free movement of charge carriers and hence constrain them to lose their energy non-radiatively, typically as heat. To determine the optimal density of defects for the proposed device in the present study, the absorber’s defect density was adjusted between 1.1 × 10^12^ and 1.1 × 10^19^ cm^− 3^. As shown in Fig. 3a, the PCE of the device dramatically dropped as the density of defects increased. This can be attributed to the increased number of recombination centres which simultaneously generate localized energy levels within the bandgap of the material. These levels act as traps for charge carriers (electrons and holes), causing them to recombine non-radiatively instead of contributing to effective processes like energy conversion in solar cells. The results obtained indicate that optimal performance is achieved when the defect density of the absorber is below 1.1 × 10^14^ cm^− 3^, which gives a PCE of 21.02% for the device. Reducing defect density minimizes non-radiative recombination, improves charge carrier lifetimes, and enhances the overall energy conversion efficiency^51^.

The fill factor (FF) is an important electrical indicator that can be utilized in estimating a solar cell’s performance^52^. A solar cell’s power conversion efficiency is closely correlated with its FF. This implies that a higher FF will generally correspond to an optimized efficiency, while an ideal FF is 1 (100%). It is often expressed as shown in Eq. 2 as the ratio of the maximum power generated by a solar device to the product of the open circuit voltage (V_oc_) and the short circuit current (J_sc_)^53^.

2 $$\:FF=\frac{{P}_{max}}{{V}_{oc}\times\:{J}_{sc}}$$

Figure 3b shows the relationship between the FF and the density of defects in the absorber, where FF decreases relative to increasing density of defects, an occurrence that can be ascribed to increased series resistance and decreased shunt resistance. However, the FF was observed to increase instantaneously at a defect level of 1.1 × 10^19^ cm^− 3^. An abrupt increase in the fill factor (FF) at high defect density is rare but can occur under specific circumstances, usually related to unique material properties or device configurations. In this case, increasing the density of defects can create localized energy states that facilitate charge transport under specific conditions, improving the FF. This is more common in materials with extraordinary electronic properties, such as perovskites^54^. Furthermore, some defects exhibit metastable traits^55^, which implies that their effect on recombination can evolve with external factors like light exposure or temperature, therefore causing a temporary enhancement of FF under special working conditions. Figure 3c and d show that the *V*_*oc*_ and *J*_*sc*_ remain relatively constant at 0.817 V and 31.4 mA cm^− 2^ respectively, when the defect level is between 10^12^ cm^− 3^ and 10^14^ cm^− 3^, but starts to decrease afterwards, dropping to approximately 0.811 V and 14 mA cm^− 2^ at defect level 10^19^ cm^− 3^ correspondingly due to factors such as the introduction of deep-level defects that act as recombination centres or changes in the device’s internal resistance.

### The influence of operational temperature on performance

**Fig. 4:**
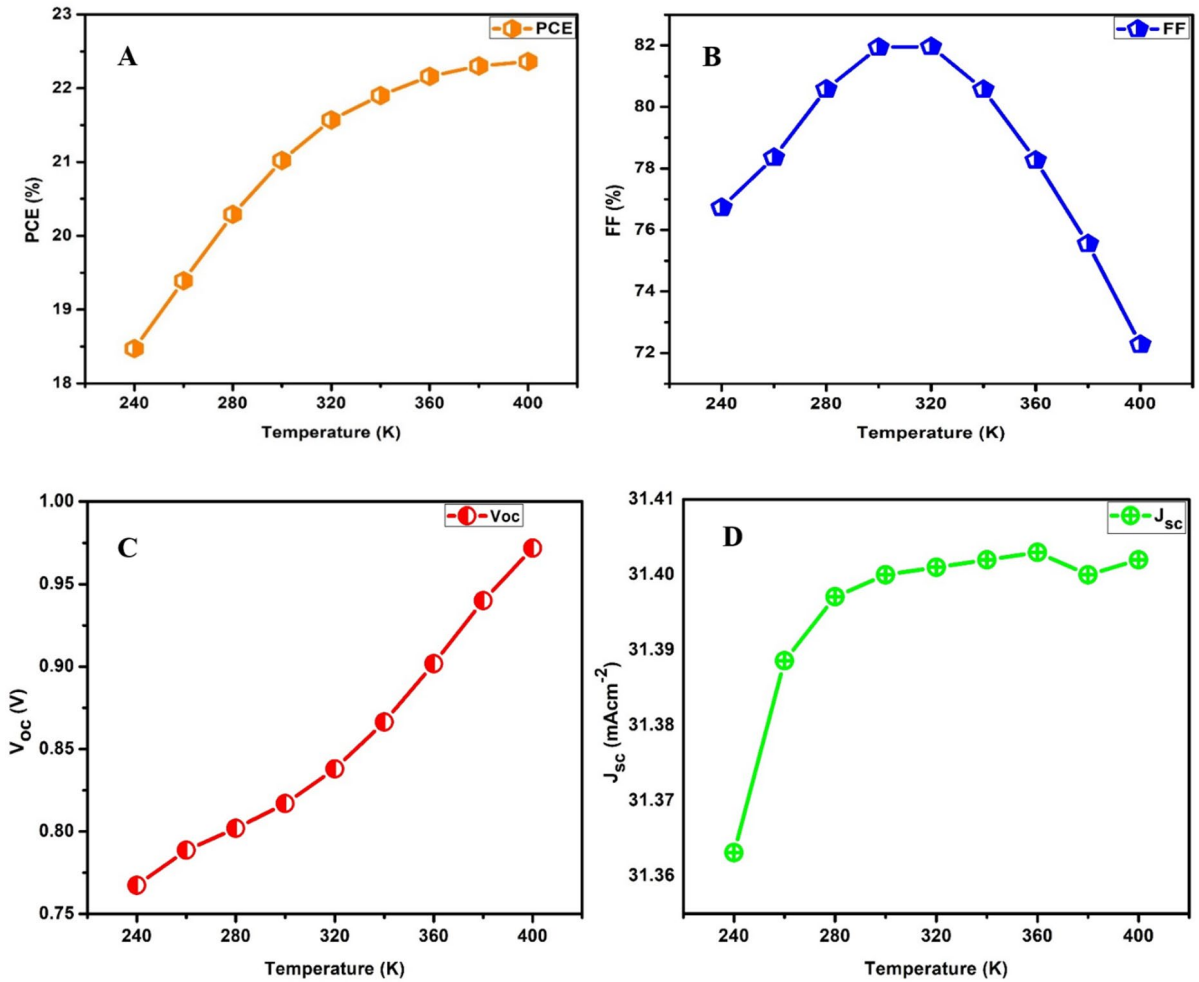
Effects of changing the operation temperature conditions of devices with CZTSe as HTLs on (**a**) PCE, (**b**) FF, (**c**) V_oc_, and (**d**) J_sc_.

Temperature-dependent measurements and analysis are important criteria for understanding the performance and stability of solar cells. Modifications in the temperature of the device can affect the charge carrier mobility and recombination rates, which are significant for improving device performance. Changes in temperature can also affect the degradation process and light absorption capacity, therefore establishing the long-term stability and efficiency of the device. Generally, Eq. 3 show the relationship between the temperature and specific photovoltaic parameters.

3 $$\:{\text{V}}_{\text{o}\text{c}}=\frac{\text{n}{\text{k}}_{\text{B}}\text{T}}{\text{q}}\text{ln}\left[\frac{{\text{J}}_{\text{s}\text{c}}}{{\text{J}}_{\text{o}}}+1\right]$$

where n is a factor, k_B_ is the Boltzmann constant, T is temperature, q is the elementary charge, and J_o_ is the dark saturation current. The term $\:\frac{\text{n}{\text{k}}_{\text{B}}\text{T}}{\text{q}}$, is the thermal voltage or flow of elementary current. The impact of the operational temperature of the proposed device from 240 to 400 K on its performance is shown in Fig. 4a. Unexpectedly, the PCE of the device with CZTSe as HTL increases as the temperature increases from 240 to 440 K. The same results were observed by Shah et al.^56^. This can be attributed to several reasons, such as improved mobility of charge carriers due to increased thermal energy, which enhances charge transport. Another reason could be due to improvement in light-trapping properties or absorption of the device at elevated temperatures. Likewise, an increase in temperature can induce diffusion of charge carriers across the interface, a behaviour that can optimize the alignment of energy levels at the HTL interface, enabling improved charge extraction. Generally, the modelled device showed that it can withstand high temperature probably because it has only inorganic components and thus formed a compact structure. Figure 4b shows modification of the FF relative to increasing temperature. In accordance with the data obtained from numerical simulation as displayed in Fig. 4b, the FF was observed to increase as the temperature increases, attaining a maximum value of 81.94% at an optimal temperature of 300 K before decreasing steadily as the temperature increases above 320 K. This behaviour suggests the relative stability of the device up to a temperature of 320 K; hence, it can function optimally within the temperature range of 240–320 K but is unstable at higher temperature domains. The extraction of electrons and holes from the photoactive layer is accomplished more efficiently at higher temperatures due to the increased mobility of the charge carriers. Conversely, elevated temperatures can equally augment recombination rates, which causes a general decrease in the fill factor, as observed in Fig. 4b. High temperatures can degrade the interfaces between different layers within the proposed device, creating charge traps that hinder the flow of charge carriers^57^. It can also cause structural changes in the materials^58^, phase transitions, or decomposition, increasing defect density and charge trapping, which leads to the formation of defect sites that act as recombination centres. While most perovskite materials exhibit a decrease in V_oc_ with increasing temperature, Fig. 4c indicates a contrasting attribute similar to a behaviour observed for a BaZrS_3_-based chalcogenide perovskite absorber using organic poly (3-hexylthiophene) as HTL^59^. The atypical increase in V_oc_ with temperature may be induced by surface effects or interface properties, which could modify charge carrier behaviour in an extraordinary manner. Furthermore, from Fig. 4d, it can be observed that as the temperature increases, the *J*_*sc*_ also increases, attaining a peak at 360 K. High temperatures can lead to an increase in the intrinsic carrier concentration, which enhances the generation of charge carriers when the solar cell is exposed to sunlight. The trend observed in Fig. 4d also suggests that there is an optimal temperature range at which *J*_*sc*_ can be maximized in the device.

### Effect of n-type doping modification on the device parameters

**Fig. 5:**
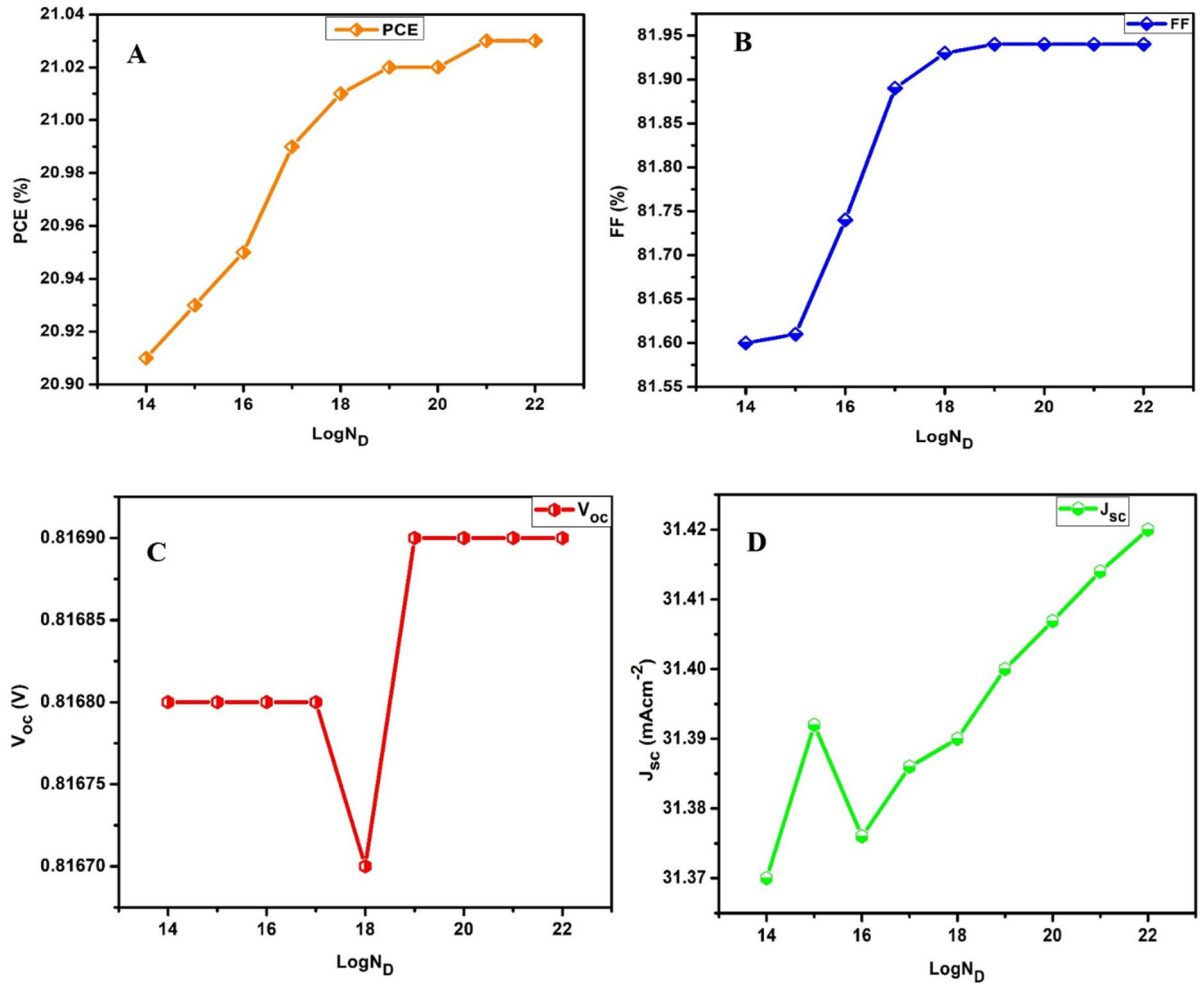
Effect of donor density of the ETL on electrical parameters of the model cell structure (**a**) PCE, (**b**) FF, (**c**) V_oc_, and (**d**) J_sc_.

Donor density optimization plays a vital role in determining the solar device performance and efficiency. An optimized donor density can improve the availability of free carriers, thereby enabling better charge collection, while insufficient donor density can limit the availability of free carriers (electrons) in the material, which leads to poorer transport efficiency and lower current output. Figure 5 shows the behaviour of proposed device parameters when the donor density is varied within a specified range. The results in Fig. 5a, b and d indicate that the PCE, FF, and J_sc_ generally improve in performance as the donor density increases. The enhanced performance can be ascribed to increased charge carrier generation and collection. However, the V_oc_ remained relatively constant until a donor density of 1.0 × 10^17^ cm^− 3^, where it drops slightly before recovering at 1.0 × 10^18^ cm^− 3^ and plateau at 1.0 × 10^19^ cm^− 3^ as seen in Fig. 5c This behaviour might indicate a phase transition or a modification in the device’s operational mechanism at a specified value of donor density. It also suggests a more complex relationship, which can be investigated further. N-type doping can be limited by dopant solubility, compensation from native defects, deep donor levels, lattice distortion at high concentrations, and temperature-dependent activation.

### Effect of p-type doping

**Fig. 6:**
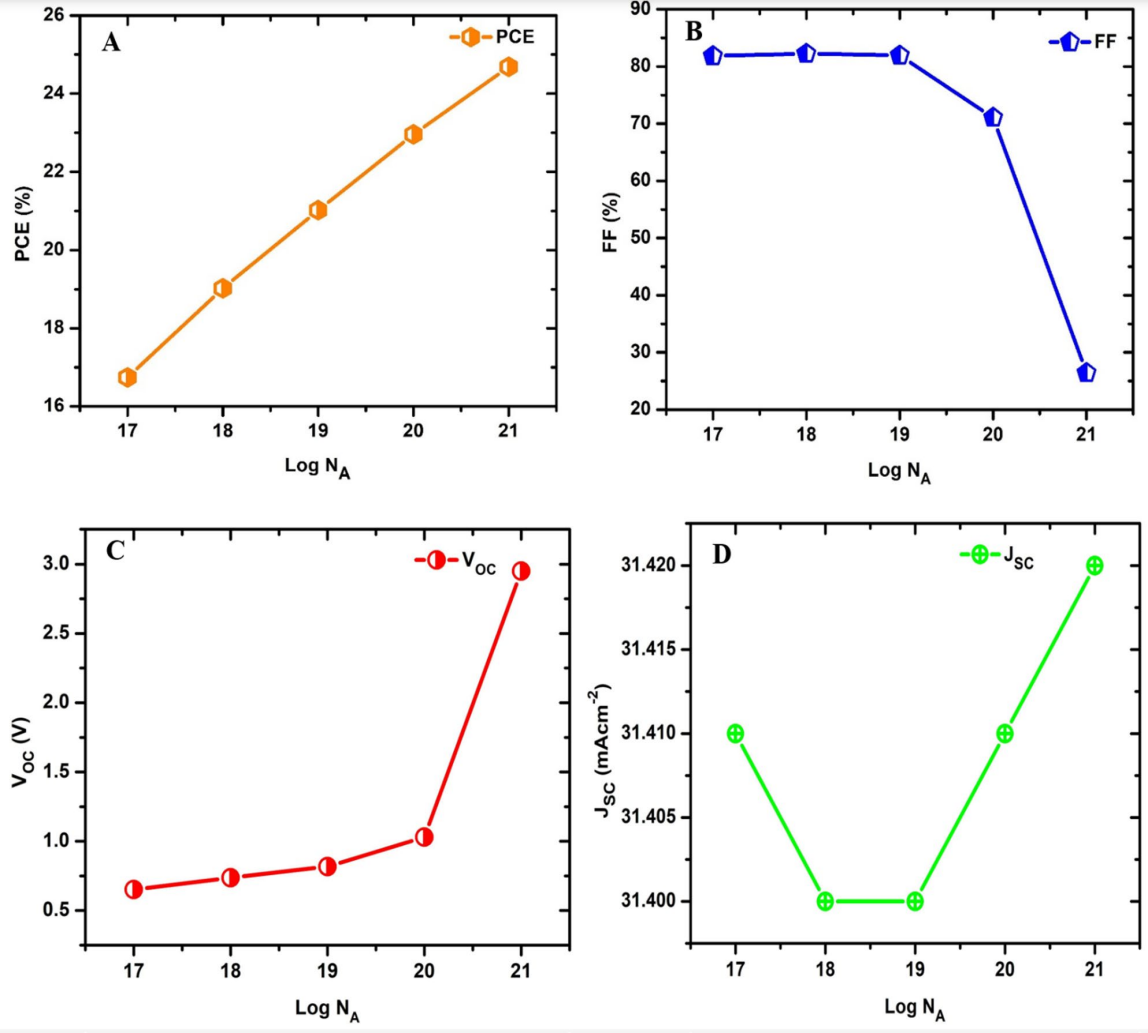
Influence of acceptor density of the HTL on electrical parameters of the model cell structure (**a**) PCE, (**b**) FF, (**c**) V_oc_, and (**d**) J_sc_.

The relationship between the electrical parameters of the proposed device while varying the acceptor density of the HTL is shown in Fig. 6. According to information obtained from the numerical simulation, the PCE was observed to increase with the logarithm of carrier concentration (Log Nₐ), as seen in Fig. 6a, which indicates a positive correlation. The PCE rises from approximately 17 to 25% as the acceptor density increases from 10^17^ to 10^21^ cm^− 3^, which indicates that higher carrier concentrations improve the solar cell’s efficiency by enhancing charge transport capabilities. The FF in Fig. 6b remains relatively stable around 80% until the acceptor density increases above 10^19^ cm^− 3^, after which it drops sharply to about 25%. The fill factor represents the quality of the solar cell, and a high FF is desired. The decrease at higher acceptor density suggests a performance drop, possibly due to increased resistance or recombination losses. The V₀_c_ shows a gradual increase with the acceptor density up to 10^20^ cm^− 3^ followed by a significant rise between 10^20^ and 10^21^ cm^− 3^.

The sharp increase at higher values of acceptor density could be related to changes in the device’s built-in potential or a reduction in recombination. The short-circuit current density in Fig. 6d was observed to exhibit a non-linear behaviour with an increase in acceptor density. This can be attributed to complex interactions between carrier generation, recombination, and transport within the device. P-type doping is often harder due to low hole mobility, donor-like defect compensation, deep acceptor levels, dopant clustering, and chemical or thermal instability. For both N and P-type doping, precise control of dopant concentration is critical, as excessive doping can increase defects and recombination, while material-specific asymmetries further complicate achieving optimal carrier densities.

### The band structure of the modelled device

**Fig. 7:**
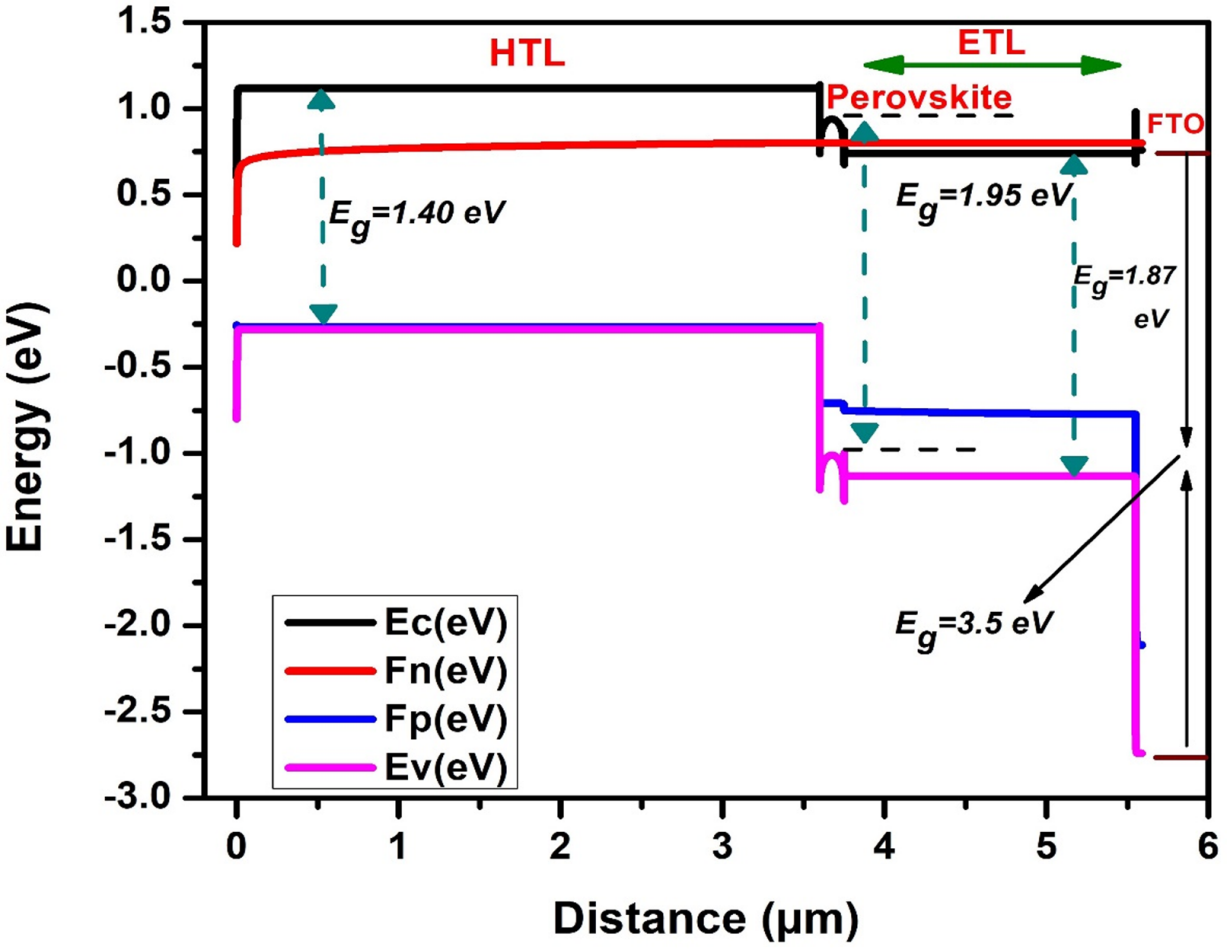
Band energy diagram of the proposed solar cell structure.

The band energy diagram for the proposed device is depicted in Fig. 7. It illustrates how the energy bands bend and aligns at the interfaces between different materials, which is fundamental towards understanding the mechanisms of charge transport and collection in the solar cell. From Fig. 7, *E*_*c*_ is the conduction band energy level, *F*_*n*_ is the quasi-Fermi level for electrons, *F*_*p*_ is the quasi-Fermi level for holes, and *E*_*v*_ is the valence band energy level. The quasi-Fermi levels for electrons and holes play a crucial role in describing the behaviour of charge carriers under non-equilibrium conditions (e.g., when a material is illuminated or subjected to an external voltage). The difference between these two quasi-Fermi levels, (*E*_*Fn*_ *- E*_*Fp*_), is directly related to the splitting of the electron and hole populations under external excitation. This difference often corresponds to the open-circuit voltage (V_oc_) in the device. Figure 7 depicts a staggered or type-II band alignment structure, which is designated by the placement of the conduction band minimum (*E*_*c*_) and valence band maximum (*E*_*v*_) of the perovskite material between those of the HTL and ETL. This arrangement facilitates effective light absorption and charge generation. It is also enabling hole extraction and blocking electron flow as a result of the higher *E*_*c*_ and lower *E*_*v*_ of the HTL compared to the absorber material.

**Fig. 8:**
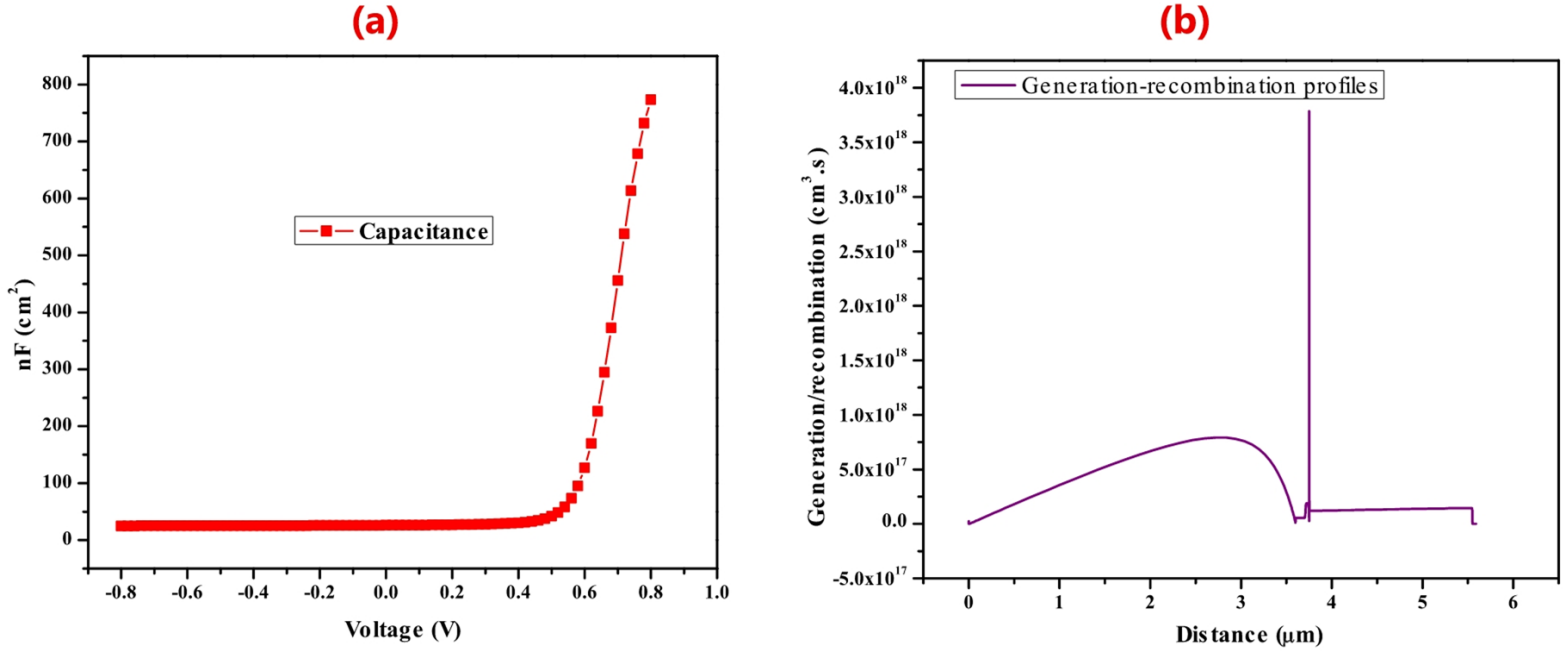
(**a**) Capacitance versus voltage and (**b**) the total generation-recombination profiles against distance curves.

The conduction band offset (CBO) between the absorber and the ETL showed a cliff-like interface^60,61^. This might have led to high recombination rates of photogenerated charges at this interface and this corroborate what was observed in the generation-recombination profile of the device (please see Fig. 8b). Nonetheless, other enhancing factors such as the fast charge transfer characteristics and better light absorption could have compensated for this and hence still high performance. On the other hand, valence band offset (VBO) between the absorber and HTL exhibited a spike -like interfaces. It has been established that the spike-like band offset is beneficial in suppressing the recombination of photogenerated charges hence at this interface there was minimal recombination^62^. The thin absorber layer in the present study was found to be cost-effective, sustainable and offers improved absorption of photons.

**Table 4:** Comparison of performance with antimony-based PSCs.

Device Architecture	PCE (%)	Study	Ref
FTO/ZnOS/Cs_3_Sb_2_I_9_/Cu_2_O/C	18.29	Simulation	^5^
FTO/TiO_2_/ Cs_3_Sb_2_I_9_/Spiro-OMeTAD/Au	12.54	Simulation	^63^
FTO/TiO_2_/ Cs_3_Sb_2_I_9_/P_3_HT/Au	2.50	Experiment	^9^
FTO/TiO_2_/MASbSI_2_/PCPDTBT/Au	3.08	Experiment	^64^
ITO/PEDOT: PSS/ Cs_3_Sb_2_I_9_/PCBM/Al	1.50	Experiment	^65^
ITO/TiO_2_/ Cs_3_Sb_2_Br_9_/Spiro-OMeTAD/Au	15.69	Simulation	^66^
FTO/TiO_2_/ MA_3_Sb_2_I_9 − X_Cl_X_/Spiro-OMeTAD/Au	3.34	Experiment	^67^
FTO/TiO_2_/ Cs_3_Sb_2_Cl_x_I_9 − x_ /Spiro-OMeTAD/Au	2.22	Experiment	^18^
FTO/WS_2_/Cs_3_Sb_2_I_9_/CZTSe/Ag	21.02	Simulation	This work

From Table 4, the gap between simulated and experimental PCEs in antimony-based perovskite solar cells arises mainly from real-world limitations not fully captured in models. Material defects, including vacancies and grain boundaries, reduce carrier lifetimes through recombination. Poor interface quality between the perovskite and transport layers introduces energy level mismatches and resistance losses. Optical effects such as reflection, parasitic absorption, and incomplete photon capture further limit current generation. Additionally, environmental instability—particularly sensitivity to moisture and oxygen—causes material degradation. Finally, variations in device architecture and fabrication processes contribute to performance discrepancies compared to idealized simulations.

### The capacitance and generation-recombination profiles

One of the significant aspects of the solar cell performance is the capacitance, and it gives insights into the device quality and general performance. Figure 8a show the graph of capacitance versus voltage of the device. As can be observed the capacitance was relatively constant at a voltage of -0.8 to about 0.5 V before experience and exponential increase after the voltage of 0.5 V. This implies that the device can store more charge at relatively high voltage. Generally, when an electron is ejected from the valence band to the conduction band, it leaves a hole (positively charge). The hole and electron can recombine leading to unavailability of mobile charges, thus leading to impeded solar cell’s performance. Essentially, there are three types of recombination in a bulk semiconductor for example solar cell device. These includes the auger, Shockley Read Hall (SRH) and radiative recombination^68^.

Figure 8b shows the total recombination profile (combination of SRH, Auger and radiative recombination) of the electron-hole pairs in the device. The generation-recombination gradually increased as a function of increasing thickness up to 3 μm. After, that it decreases for a distance between 3 to about 3.8 μm before experiencing a very sharp increase at the interface between the absorber and ETL (WS_2_) an indication that at the interface both the generation and recombination process was dominant. At the ETL material the generation-recombination was apparently constant implying that no generation or recombination was taking place at this part of the device.

## Conclusion

The analysis of antimony-based halide perovskite solar cells using kesterite as hole transport material has been investigated in the present study through numerical simulations with a focus on performance optimization. The n-i-p solar device with architecture FTO/WS_2_/Cs_3_Sb_2_I_9_/CZTSe/Ag was theoretically modelled with WS_2_ as electron transport material. The influence of basic parameters on the proposed device, such as the effect of changing the doping density of the HTL, the density of defects in the absorber layer, and the device operational temperature, has been reported in this work for optimal performance of the device. Results obtained from the simulation indicate that the proposed device has prospects of light absorption within the visible range of the electromagnetic spectrum. The temperature-dependent measurements and analysis also suggest that the device can function optimally within the temperature range of 240–320 K but is unstable at higher temperature domains. The short-circuit current density (*J*_*sc*_) was observed to exhibit a non-linear behaviour with an increase in acceptor density, which can be attributed to complex interactions between carrier generation, recombination, and transport within the device. An optimal power conversion efficiency (PCE) of up to 21.02% with a fill factor (FF) of 81.94% was achieved for the proposed device configuration, while the band energy diagram for the proposed device depicted a type II band alignment structure. The estimated *V*_*oc*_ and *J*_*sc*_ values of 0.812 V and 31.40 mAcm^− 2^, respectively was obtained. It is important to point out that the SCAPS-1D only work in one dimension and thus the results are only restricted to one dimensionality, yet the device is in 3 dimensions, hence this is one of the limitations of the simulator. Moreover, the simulated results might be exaggerated because the model doesn’t factor in environmental aspects such as moisture, temperature and degradation of devices which are significant during actual experimental fabrication of the devices. Nonetheless, the simulated results suggest that Cs_3_Sb_2_I_9_ can be a promising absorber material for the development of lead-free perovskite solar cells.

## Data Availability

All data generated or analysed during this study are included in the manuscript.
